# Assessment of the nail contamination with soil-transmitted helminths in schoolchildren in Jimma Town, Ethiopia

**DOI:** 10.1371/journal.pone.0268792

**Published:** 2022-06-29

**Authors:** Bamlaku Tadege, Zeleke Mekonnen, Daniel Dana, Abebaw Tiruneh, Bizuwarek Sharew, Eden Dereje, Eskindir Loha, Mio Ayana, Bruno Levecke

**Affiliations:** 1 School of Medical Laboratory Sciences, Hawassa University, Hawassa, Ethiopia; 2 School of Medical Laboratory Sciences, Jimma University, Jimma, Ethiopia; 3 Department of Translational Physiology, Infectiology and Public Health, Ghent University, Merelbeke, Belgium; 4 Molecular Biology and NTDs Research Center, Jimma University, Jimma, Ethiopia; 5 Chr. Michelsen Institute, Bergen, Norway; 6 Centre for International Health, University of Bergen, Bergen, Norway; Dokkyo Medical University, JAPAN

## Abstract

**Background:**

Large-scale deworming programs have been successful in reducing the burden of disease due to soil-transmitted helminth (STH; *Ascaris lumbricloides*, *Trichuris trichiura* and hookworm) infections, but re-infection in absence of other measures is unavoidable. We assessed the role of nail contamination as a source of infection with the goal to evaluate the potential of nail clipping as a simple measure to further reduce STH-attributable morbidity.

**Methods:**

A cross-sectional study was conducted in Jimma Town (Ethiopia). Both stool samples and clipped nails were collected from 600 schoolchildren and microscopically screened for the presence of STHs. We also interviewed the children to gain insights into their hygiene practices. Subsequently, we explored any associations between infection, nail contamination and personal hygiene.

**Results:**

Any STH infections were observed in 24.3% of the children (*A*. *lumbricoides*: 18.5%; *T*. *trichiura*: 9.8%; hookworm: 0.5%). The intensity of the infections was mainly low, only in a few cases a moderate-to-heavy intensity infection was observed (*A*. *lumbricoides*: 4.3%; *T*. *trichiura*: 0.2%). Other helminth species observed were *Schistosoma mansoni* (5.0%), *Hymenolepis nana* (2.7%), *Taenia* spp. and *Enterobius vermicularis* (<1.0%). The analysis of the nail material revealed the presence of *A*. *lumbricoides* (1.7%), *Taenia* spp. (1.0%), *T*. *trichiura* (0.5%), *E*. *vermicularis* (0.5%) and *H*. *nana* (0.2%). The odds of infection with any STH increased as the frequency of trimming decreased. The odds of nail contamination with any STH and *A*. *lumbricoides* were higher for younger children.

**Conclusions:**

The presence of helminth eggs under the nails of children highlights a poor personal hygiene. The association between any STH infection and frequency of nail trimming needs to be explored in an intervention study. The recent prevalence of any STH infections indicated that scaling down of the frequency of deworming is justified but that STH is still a public health problem.

## Introduction

Soil-transmitted helminths (STHs) are a group of intestinal worms that are transmitted through the uptake of infectious stages residing in the environment (often soil—referring to their common name [1]). The four main STH species that infect humans are *Ascaris lumbricoides* (giant roundworm), *Trichuris trichiura* (whipworm), and the two hookworms *Ancylostoma duodenale* and *Necator americanus*. It is estimated that over one-fifth of the world population is infected with at least one of these STH species, resulting in an estimated global disease burden of 3.4 million disability-adjusted life years (DALYs) [2, 3]. The STH-attributable morbidity is associated with moderate-to-heavy intensity infections, and mainly affects children and women of childbearing age [4, 5], causing malnutrition, anaemia, stunted growth, and both impaired physical and cognitive development [6, 7].

The World Health Organization (WHO) recommends three interventions to control this STH-attributable morbidity, including (i) periodical administration of anthelmintic drugs to kill the worms, and both (ii) health education and (iii) improved water, hygiene, and sanitation (WASH) to prevent re-infection [8]. Up-to-date current control strategies have mainly been focusing on large-scale deworming programs during which at-risk populations are treated once or twice per year with a single oral dose of albendazole (400 mg) or mebendazole (500 mg) [9]. Recently, these programs have reached a treatment coverage that is unprecedented in history (coverage of children increased from 32.6% in 2012 [10] to 57.6% in 2019 [11]. Although these large-scale deworming programs have been successful in reducing the disease burden [12], re-infection in absence of other supplementary intervention measures is unavoidable [13, 14]. Although, it is known that WASH and disease awareness will be essential to further control and eventually eliminate STH infections, these measures require important financial and political commitments, and are often faced with scepticism from the community [15–18].

Moreover, it remains unclear where and how re-infection occurs, which further impedes the development of targeted and perhaps more cost-effective control strategies. For example, a variety of studies already highlighted the presence of STH life stages around households [19, 20], school compounds (playgrounds [21, 22] and latrines [21, 23]) and markets [24], but the implementation of measures to reduce this environmental contamination will not be trivial. Untrimmed finger nails are another potential source of STH infections, but regularly clipping of nails may potentially prevent (re-)infections. Indeed, various studies targeting schoolchildren have indicated nail contamination with STHs, reporting prevalence up to ~20% for *A*. *lumbricoides*, ~13% for *T*. *trichiura* [25] and ~4% for hookworms [26]. The present study assessed the presence of STH eggs under finger nails of schoolchildren at Jimma Town (Ethiopia) and whether factors, such as presence of STH eggs under finger nails, poor personal hygiene or other demographic contribute to STH infections.

## Materials and methods

### Study design

Between October and December 2020, we screened children from ten primary governmental schools across 6 kebeles (lowest administrative level) at Jimma Town (Southwest Ethiopia). The selection of the schools was based on the presence of the age groups of interest (5 to 18 years) and their participation in previous studies [27–29]. At each school, 60 children were sampled (30 children between the age 5 and 9, and 30 children between the age of 14 and 18), resulting in a total of 600 children. This sampling scheme was also applied in previous studies [27, 28]. Each child that consented to participate in the study was asked to deliver a fresh stool of at least 2 g in a clean and labelled stool container. Stool samples were processed by a single Kato-Katz thick smear, the current diagnostic standard in deworming programs for STHs [30]. In addition, nails from all 10 fingers were clipped from each child using a sterile nail clipper (one clipper per child). All nails were put in a labelled container that contained 5 ml of normal saline. Finally, we interviewed children with the assistance of their parents/guardians to gain insights into their personal hygiene practices, including but not limited to their nail trimming status/habit and hand washing practices. We attached the English version of the questionnaire as **S1 File**.

### Parasitological examination

Stool samples were processed applying a single Kato-Katz thick smear as previously described [30]. The smears were microscopically examined for the presence of STH eggs, and the intensity of infection was estimated through fecal egg counts (FECs; expressed as eggs per gram of stool (EPG)). The presence of eggs of other intestinal helminths were also recorded, but we did not count the number of eggs.

The containers containing the nails were vigorously shaken to dislodge the material from the nails. Subsequently, the suspension was transferred into a new 15 ml conical tubes. To ensure that all material/content was transferred, the original container was rinsed twice with 5 ml normal saline. Subsequently, the 15 ml tube was vigorously shaken and centrifuged at 2,500 *g* for 3 minutes [31]. After discarding the supernatant, the sediment was transferred to a microscopic slide and microscopically examined for the presence of helminth eggs/larvae. As part of a quality control process, 10% of the Kato-Katz thick smears (stool samples) were re-examined by an experienced parasitologist.

### Statistical data analysis

We reported the patterns of infections (stool samples) and contamination (nails) with STHs, data related to children’s hygiene, and the associations between infection and personal hygiene. For both the infections and the contamination with STHs, we determined the prevalence (proportion of samples in which eggs were detected) of any STH, and each STH species separately. For the STH infections, we also determined the prevalence of low and moderate-to-heavy infections intensities. For this, we applied the WHO classification criteria based on FECs by Kato-Katz thick smear [32]. For all other helminths, we only reported the prevalence. Standard descriptive statistics were used to report the outcome of the interviews on personal hygiene.

Multivariable logistic regression was used to model the presence of eggs in stool and under the nails. The potential predictive variables included in the models for infections (stool) were ‘age’ (2 levels: age 5–9 *vs*. age 14–18), ‘sex’ (2 levels: female *vs*. male), ‘finger sucking behaviour (2 levels: yes *vs*. no), ‘frequency of finger nail trimming’ (ordinal variable with 3 levels: once per week; once 2–3 weeks; when they are long enough), ‘trimming material’ (2 levels: teeth *vs*. razor/clipper), ‘hand washing before eating’ (2 levels: sometimes *vs*. always), ‘hand washing after toilet’ (2 levels: sometimes *vs*. always), ‘playing games that involve contact with soil’ (2 levels: yes *vs*. no) and ‘nail contamination’ (2 levels: yes *vs*. no). For the models for nail contamination, the same predictive variables were considered, except for ‘hand washing before eating’ and ‘nail contamination’, which was excluded from the model and replaced by ‘presence of eggs in stool’ (2 levels: yes *vs*. no). The selection of these variables was mainly based on both the prior knowledge of potential risk factors of infection and statistical aspects (e.g., minimum lambda using least absolute shrinkage and selection operator (LASSO) for logit model (to some extent) and absence of multicollinearity were considered). Hosmer-Lemeshow chi-square value was used to ascertain goodness-of-fit of the models. We build models for any STH, *A*. *lumbricoides* and *T*. *trichiura* eggs in stool, and any STH and *A*. *lumbricoides* under the nails only. Due to the small sample size and few cases, we did not consider models for presence of eggs or nail contamination for the other helminth species.

### Ethics statements

The study protocol was approved by the Institutional Review Board of Jimma University, Ethiopia (reference number: IHRPGD/466/2020). The school authorities, teachers, parents and the children were informed about the purpose and procedures of the study. A written informed consent was obtained from the parents/guardians. An additional separate written consent was secured from children older than 12 years. Consent forms were prepared in English, and subsequently translated into the commonly used local languages (Afaan Oromo). Only those children who were willing to participate and whose parents or guardians signed the written informed consent form were included in the study. All individuals infected with any STH were administered with a single oral dose of 400 mg albendazole [9]. For children infected with *Schistosoma* or cestodes, we recommended their parents to take them to the clinic for an anthelmintic treatment.

## Results

### The pattern of STH infections and nail contamination with STH eggs

A total of 600 stool and 600 finger nail samples were collected from an equal number of schoolchildren. The study population consisted of 313 (52.2%) girls and 287 (47.8%) boys. Overall, any STH eggs were observed in 24.3% of the stool samples (146/600), *A*. *lumbricoides* being the most prevalent (18.5%; 111/600). *T*. *trichiura* infections were observed in 9.8% (59/600) and hookworm infections in 0.5% (3/600) of the subjects. The intensity of the infections was low for most of the subjects, only in a few cases a moderate-to-heavy intensity infection was observed (*A*. *lumbricoides*: 4.3%, n = 26), *T*. *trichiura*: 0.2% n = 1). Other helminth species observed were *Schistosoma mansoni* (5.0%, n = 30), *Hymenolepis nana* (2.7%, n = 16), *Taenia* spp. (0.8%, n = 5) and *Enterobius vermicularis* (0.2%, n = 1).

**Fig 1:**
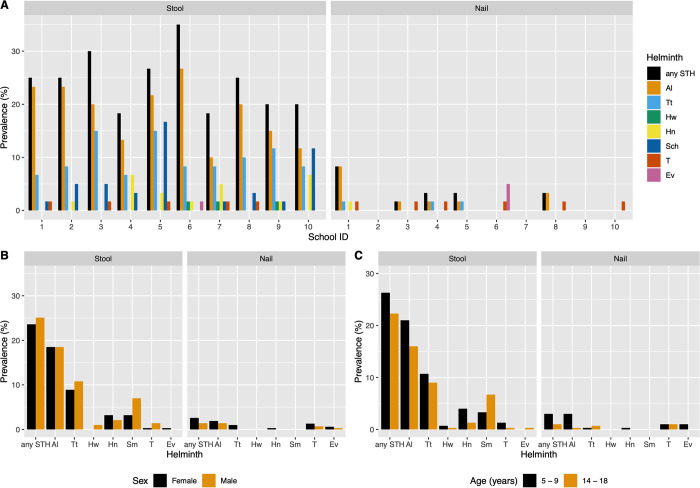
The prevalence of helminth eggs in stool and under finger nails across 600 schoolchildren. The bar plots represent the prevalence of helminth eggs in stool and under finger nails across 10 school (**Panel A**), 2 sexes (**Panel B**) and 2 age groups (**Panel C**). **Any STH**: any soil-transmitted helminth, **Al**: *Ascaris lumbricoides*, **Tt**: *Trichuris trichiura*, **Hw**: hookworm, **Hn**: *Hymenolepis nana*, **Sch**: *Schistosoma mansoni*, **T**: *Taenia* spp., **Ev**: *Enterobius vermicularis*.

For each of the different helminth species, there was a large variation across schools (**Fig 1A**). Both *A*. *lumbricoides* and *T*. *trichiura* were observed in all schools, with the school prevalence ranging from 10.0 to 26.7% for *A*. *lumbricoides*, and from 6.7% to 15.0% for *T*. *trichiura*. The prevalence of any STH infection ranged from 18.3% to 35.0%. Except for one school (School #6), infections with *S*. *mansoni* was observed in all schools. The remaining helminths were observed in 7 schools (*H*. *nana*), 5 schools (*Taenia* spp.), 3 schools (hookworm) and 1 school (*E*. *vermicularis*). Except for *S*. *mansoni* (16.7%) and *H*. *nana* (6.7%), the school prevalence did not exceed 2.0% for the other helminths. No distinct differences in prevalence across both sexes were observed (*A*. *lumbricoides*: 18.5% *vs*. 18.5%; *T*. *trichiura*: 10.8% *vs*. 8.9%; **Fig 1B**). For *S*. *mansoni*, boys were more infected than girls (7.0% *vs*. 3.2%). Except for both *S*. *mansoni* for which children aged 5–9 years (3.3%) were generally less infected than those aged 14–18 years (6.7%), and *A*. *lumbricoides* for which children aged 5–9 years were more infected (21.0% *vs*. 16.0%; **Fig 1C**), we did not observe major difference in prevalence across the two age groups.

The analysis of the nail materials revealed the presence of five helminth species (*A*. *lumbricoides*, *T*. *trichiura*, *Taenia* spp, *E*. *vermicularis* and *H*. *nana*). The prevalence of any STH equalled 2.0% (12/600). The species-specific prevalence for the five different helminth species detected were 1.7% for *A*. *lumbricoides* (10/600), 1.0% for *Taenia* spp. (6/600), 0.5% (3/600) for *E*. *vermicularis* and *T*. *trichiura*, and 0.2% for *H*. *nana* (1/600). For each of the different helminth species, there was a considerable variation across schools (**Fig 1A**). Both *A*. *lumbricoides* and *T*. *trichiura* were observed in 5 and 3 schools, respectively, with the school prevalence ranging from 1.7–8.3% for *A*. *lumbricoides*, and 1.7% for *T*. *trichiura* for each school. Other helminths were observed in 6 schools (*Taenia* spp.) and 1 school (*E*. *vermicularis* and *H*. *nana*). For each of these helminths the prevalence did not exceed 5.0%. Distinct differences in helminth specific prevalence were absent across both sexes (*A*. *lumbricoides*: 1.4% *vs*. 1.9%, *T*. *trichiura*: 0.0% *vs*. 1.0% (**Fig 1B**). For *A*. *lumbricoides*, the nails of children aged 5–9 years were slightly more contaminated than those aged 14–18 years (3.0% *vs*. 0.3%). For the remaining helminths, we did not observe a distinct difference in the prevalence (**Fig 1B**). The egg counts varied from 1–10 for both *A*. *lumbricoides* and *T*. *trichiura*. **S1** and **S2 Tables** provide a numerical summary of the prevalence of helminth eggs in stool and under finger nails across the 10 schools, both sexes and the 2 age groups.

### Personal hygiene

The outcome of the questionnaire on the hygiene of the children is summarized in more detail in **S3 Table.** Except for 19 subjects (3.2%), who refused/unable to reply to the questions related to the frequency of trimming and the material used to trim their nails, all subjects responded to all questions. Only a minority of the children had a finger sucking behaviour (42/600; 7.0%). Nail trimming was practiced by almost all interviewed children (580/600; 96.7%). Children mainly trimmed their nails when they were long enough (315/581; 54.2%) or every 2 weeks (237/581; 40.8%). To trim the nails, either a nail clipper (385/581; 66.3%) or a razor (179/581; 30.8%) was used. Only few subjects used their teeth (17/581; 2.9%). With a few exceptions (43/600; 7.2%), all children responded that they always washed their hands before eating food. To wash their hands, soap was always used by 273 (45.5%) children. Hand washing was less practiced after toilet usage, children washed their hands either always (379/600; 63.2%) or sometimes (221/600; 36.8%). From all children, 197 (32.8%) always used soap, 364 (60.7%) sometimes used soap and the remaining 39 (6.5%) never used soap. A total of 253 children (42.2%) indicated that they played games that involve contact between hands and soil (e.g., teter, kelbosh and segno-maksegno). Finally, the questionnaire indicated that 467 (77.8%) children believed that untrimmed finger nail may be source of STH infections.

### The associations between infection and personal hygiene, age and sex

The output of the multivariate regression analysis is summarized in **Table 1**. The odds of infection with any STH increased by 1.6 (95% CI: 1.1–2.3; *p* = 0.011) as the frequency of trimming decreased. There was also a significant association between any STH nail contamination and age. The adjusted odds of nail contamination with any STH being 4.2 times greater (95% CI: 1.04–17.0; *p* = 0.044) for the age group of 5–9 years compared to the odds for the age group of 14–18 years. The odds of nail contamination with *A*. *lumbricoides* eggs was 11-fold (95% CI:1.3–92.7; *p* = 0.027) greater than those for the age group of 5–9 years compared to children of 14–18 years old. For the remaining variables there was no evidence of significant association.

**Table 1:** Association between infections with soil-transmitted helminth infections, and sex, age and personal hygiene. This table reports the adjusted odds ratios (95% confidence interval) derived from the multi-variable regression models. Significant associations (*p* <0.05) are indicated in bold. ^§^Reference: 14–18 years old; ^¥^Frequency of trimming in decreasing ordinal order (once per week → once per 2 to 3 weeks→ when it is long enough); ^€^Reference: using either razor blade or nail clipper.

Variables	Any STH		*Ascaris lumbricoides*		*Trichuris trichiura*
	Stool	Nail	Stool	Nail	Stool
Sex (male)	0.98 (0.7–1.5)	0.5 (0.1–1.7)	0.9 (0.6–1.4)	0.7 (0.2–2.5)	1.2 (0.7–2.1)
Age (5–9 years) ^§^	1.2 (0.8–1.8)	**4.2 (1.04–17.0)**	1.3 (0.9–2.1)	**11.0 (1.3–92.7)**	1.3 (0.7–2.7)
Finger sucking	0.7 (0.3–1.6)	1.1 (0.1–9.7)	0.6 (0.2–1.5)	1.5 (0.2–13.5)	0.9 (0.3–2.8)
Nail trimming^¥^	**1.6 (1.1–2.3)**	0.7 (0.3–2.1)	1.5 (0.98–2.2)	0.4 (0.1–1.4)	1.7 (0.9–2.9)
Trimming material (teeth)^€^	0.7 (0.2–2.4)	3.6 (0.4–32.7)	1.01 (0.3–3.7)	4.4 (0.4–46.8)	0.6 (0.1–4.5)
Hand washing before eating	1.3 (0.7–2.7)	_	1.6 (0.8–3.4)	_	0.7 (0.2–2.3)
Hand washing after toilet	1.05 (0.7–1.6)	0.8 (0.2–2.9)	1.2 (0.8 1.9)	1.1 (0.3–4.8)	0.8 (0.5–1.5)
Playing game that involves sloil (yes)	1.2 (0,8–1.7)	0.5 (0.2–1.8)	1.2 (0.8–1.9)	0.7 (0.2–2.5)	0.9 (0.5–1.7)
Nail contamination	0.6 (0.2–3.0)	`_	0.5 (0.1–3.9)	_	4.6 (0.4–54.8)
Eggs in stool	_	0.6 (0.1–3.0)	_	0.4 (0.1–3.6)	_

## Discussion

Although large-scale deworming programs have been successful in reducing the STH-attributable morbidity, reinfection in the absence of other supplementary intervention measures is unavoidable. We assessed the role of nail contamination as a source of infection with the goal to evaluate the potential of nail clipping as a simple measure to further reduce STH-attributable morbidity.

### Nail contamination underscores poor personal hygiene

We observed eggs of *A*. *lumbricoides* (1.7%), *T*. *trichiura* (0.5%), *Taenia* spp. (1.0%) and *E*. *vermicularis* (0.5%) under the nails of 19 (3.2%) children, underscoring the poor personal hygiene. These helminths were also observed under the nails of children in Pakistan (30/300; 10% [31]), in Iraq (18/103; 17.5% [33]) and Nigeria (20/200: 10% [34]). Hookworm eggs were also observed in Nigeria (21/325: 6.5% [25]), but not in the present study. Generally, the presence of helminths in our study can be explained by either the presence of patent infections or the biology of the helminths. Indeed, the two most prevalent helminth species under the nails are also those that cause the most patent infections in the children (*A*. *lumbricoides*: 18.5%; *T*. *trichiura*: 9.8%). Although patent *Taenia* spp. (1.0%) and *E*. *vermicularis* (0.5%) infections were only observed in a few cases, each of these worm cause itching around the anus, which potentially leads to eggs under the nails. In case of *Taenia* spp. (*T*. *saginata*), proglottids may actively leave the host [35, 36]. For *E*. *vermicularis*, the itching is caused by the adult female worms migrating towards the anus to deposit eggs at the perianal region [37]. The absence of the other helminths for which patent infections were observed but that were not detected under the nails, were either due to the low level of patent infections (*S*. *mansoni*; 5.0%; hookworm; 0.5%) or to the absence of any stimulants that triggers contact with the anus. Another cause of these absence might be the poor diagnostic performance of the modified wet smear [31]. The association between nail contamination and the age of the schoolchildren (children of 5–9 years old more likely to have STH eggs under the nails compared to school children of 14–18 years old) is not expected, and can be explained by differences in personal hygiene [38, 39].

### Trimming of nails holds promise as a simple additional prevention measure to avoid STH

The children interviewed in the present study mainly trimmed their nails when they were long enough (54.2%). This finding is considerably less than a previous study in India where schoolchildren trimmed their nails once a week (73%) [40]. This difference in frequency is might be due to a difference in the age of the study population, and hence reflecting differences in personal hygiene [41]. Indeed, while the study in India involved schoolchildren from grade 6 to 8, we included subjects between grade 3 and 8. We observed an association between the frequency of nail trimming and the presence of both any STH and *A*. *lumbricoides* infections in schoolchildren. At this stage it remains unclear whether a higher frequency of nail trimming will indeed prevent STH infection (causal affect) or whether it is a proxy of general hygiene (or any other factor that is associated with both the frequency of nail trimming and any STH/*A*. *lumbricoides* infections). To further explore the preventive impact of nail trimming on transmission of STH in large-scale deworming programs, more evidence will need to be gathered. Moreover, it might be of interest to assess the synergetic effect between trimming of nails and hand washing (one could hypothesize that the preventive impact of handwashing might be higher when nails are trimmed). This may require a longitudinal clustered randomized controlled study during which the rate of (re-)infection is compared across three intervention arms (one arm where both trimming nails and hand washing are promoted; one arm where only trimming nails is promoted; and one arm where none of these measures are promoted). A particular challenge will be to determine the study period and the required sample size. This is because the additional effect of this intervention might be small (adjusted odds ratio = 1.6 (1.1–2.3)). Meanwhile, it is important to note that trimming of nails is not meant to replace handwashing, rather it should be promoted has as a simple additional prevention measure to avoid STH.

### Large scale deworming programs reduce STH-attributable morbidity

Shortly following a nation-wide survey to map the distribution of STH infections (2013 and 2015), Ethiopia rolled out this national deworming campaigns targeting schoolchildren. At that time, our study area (Jimma Town) was eligible for distribution of drugs every 6 months (prevalence of any STH was 57.7% for infection of any intensity and 12.3% for moderate-to-heavy intensity infections [27]; **S4 Table**). Between 2015–2020, over 100 million doses of benzimidazole drugs were distributed in Ethiopia, resulting in a program coverage of 75.0% in 2018 [42]. Our results underscore that the prevalence of any STH infections both of any (23.4%) and moderate-to-heavy intensity infections (4.5%) have significantly reduced. These findings are not unexpected and are in line with previous studies [43–47]. Although there is no doubt that the national deworming program has contributed to this significant reduction in the STH-attributable, there are also a number of other aspects that need to be considered. First, Jimma Town has made significant investments to improve the WASH facilities at the schools, which may have also contributed to reduce transmission STH of infections. Second, this trial was conducted in midst of COVID-19 pandemic, and hand washing was stimulated at school to prevent COVID-19 by both health education and the installation of hand washing facilities with clean soap and water. Based on these most up to date prevalence data on any STH infections, the frequency of anthelminthic drug distribution can be reduced to once a year [48], resulting in a more efficient allocation of already scarcely available funds. Since that the prevalence of moderate-to-heavy intensity infections exceeds the 2% target, we cannot claim that STH has been eliminated as public health problem.

## Conclusions

The presence of helminth eggs under the children’s nails highlights poor hygiene, particularly in young children. Although an association was observed between any STH infection, underscoring that frequently trimming of nails holds promise as a simple prevention measure to avoid STH infection. This needs to be further explored in an intervention study. The recent prevalence of any STH infections indicated that scaling down of the frequency of deworming is justified but that STH is still a public health problem.

## Supporting information

S1 Table The prevalence of helminth eggs in stool of 600 schoolchildren, Jimma Town (Ethiopia). (DOCX)

S2 Table The prevalence of helminth eggs under the finger nails of 600 schoolchildren, Jimma Town (Ethiopia). (DOCX)

S3 Table The personal hygiene practice among 600 schoolchildren from 10 governmental schools in Jimma Town (Ethiopia). (DOCX)

S4 Table The long-term impact of the national deworming program on STH infections in Jimma Town (Ethiopia). This table summarizes the prevalence of soil-transmitted helminth infections (STH; *Ascaris lumbricoides*, *Trichuris trichiura* and hookworm) of any intensity and moderate-to-heavy intensity (MHI) across 3 cross-sectional surveys (2015, 2018 and 2020). In each survey, 600 different subjects from ten governmental schools were included.
(DOCX)

S1 Data The original raw data. (XLSX)

S1 File The English version of the questionnaire on personal hygiene practices. (DOCX)
