# Supplementary material for: Assessment of the nail contamination with soil-transmitted helminths in schoolchildren in Jimma Town, Ethiopia
Source: PLoS One. 2022 Jun 29;17(6):e0268792. doi: 10.1371/journal.pone.0268792 (PMC9242460; doi:10.1371/journal.pone.0268792)
Supplement: S1 Table — (DOCX) [file pone.0268792.s001.docx]

**S1 Table. The prevalence of helminth eggs in stool of 600 school children, Jimma Town (Ethiopia).**

|  | |  | **N** | | **Number of cases (%)** | | | | | | | | | | | | | |  | |  |
| --- | --- | --- | --- | --- | --- | --- | --- | --- | --- | --- | --- | --- | --- | --- | --- | --- | --- | --- | --- | --- | --- |
|  |  |  |  |  | Any STH | | *Ascaris* | | *Trichuris* | | Hookworm | | *Schistosoma* | | *Hymenolepis* | | *Taenia* | | *Enterobius* | |  |
| School | | | | | | | | | | | | | | | | | | |  | |  |
|  | 1 | | | 60 | | 15 (25.0) | | 14 (23.3) | | 4 (6.7) | | 0 | | 1 (1.7) | | 0 | | 1 (1.7) | | 0 | |
|  | 2 | | | 60 | | 15 (25.0) | | 14 (23.3) | | 5 (8.3) | | 0 | | 3 (5.0) | | 1 (1.7) | | 0 | | 0 | |
|  | 3 | | | 60 | | 18 (30.0) | | 12 (20.0) | | 9 (15.0) | | 0 | | 3 (5.0) | | 0 | | 1 (1.7) | | 0 | |
|  | 4 | | | 60 | | 11 (18.3) | | 8 (13.3) | | 4 (6.7) | | 0 | | 2 (3.3) | | 4 (6.7) | | 0 | | 0 | |
|  | 5 | | | 60 | | 16 (26.7) | | 13 (21.7) | | 9 (15.0) | | 0 | | 10 (16.7) | | 2 (3.3) | | 1 (1.7) | | 0 | |
|  | 6 | | | 60 | | 21 (35.0) | | 16 (26.7) | | 5 (8.3) | | 1 (1.7) | | 0 | | 1 (1.7) | | 0 | | 1 (1.7) | |
|  | 7 | | | 60 | | 11 (18.3) | | 6 (10.0) | | 5 (8.3) | | 1 (1.7) | | 1 (1.7) | | 3 (5.0) | | 1 (1.7) | | 0 | |
|  | 8 | | | 60 | | 15 (25.0) | | 12 (20.0) | | 6 (10.0) | | 0 | | 2 (3.3) | | 0 | | 1 (1.7) | | 0 | |
|  | 9 | | | 60 | | 12 (20.0) | | 9 (15.0) | | 7 (11.7) | | 1 (1.7) | | 1 (1.7) | | 1 (1.7) | | 0 | | 0 | |
|  | 10 | | | 60 | | 12 (20.0) | | 7 (11.7) | | 5 (8.3) | | 0 | | 7 (11.7) | | 4 (6.7) | | 0 | | 0 | |
| Sex | | | | | | | | | | | | | | | | | | |  | |  |
|  | Female | | | 313 | | 74 (23.6) | | 58 (18.5) | | 28 (8.9) | | 0 | | 10 (3.2) | | 10 (3.2) | | 4 (1.4) | | 0 | |
|  | Male | | | 287 | | 72 (25.1) | | 53 (18.5) | | 31 (10.8) | | 3 (1.0) | | 20 (7) | | 6 (2.1) | | 1 (0.3) | | 1 | |
| Age group (years) | | | | | | | | | | | | | | | | | | |  | |  |
|  | 5 – 9 | | | 300 | | 79 (26.3) | | 63 (21.0) | | 32 (10.7) | | 2 (0.7) | | 10 (3.3) | | 12 (4.0) | | 4 (1.3) | | 0 | |
|  | 14 – 18 | | | 300 | | 67 (22.3) | | 48 (16.0) | | 27 (9.0) | | 1 (0.3) | | 20 (6.7) | | 4 (1.3) | | 1 (0.3) | | 1 (0.3) | |
| **Total** | | | **600** | | **146 (24.3)** | | **111 (18.5)** | | **59 (9.8)** | | **3 (0.5)** | | **30 (5.0)** | | **16 (2.7)** | | **5 (0.8)** | | **1 (0.2)** | |  |
