# Supplementary material for: Assessment of the nail contamination with soil-transmitted helminths in schoolchildren in Jimma Town, Ethiopia
Source: PLoS One. 2022 Jun 29;17(6):e0268792. doi: 10.1371/journal.pone.0268792 (PMC9242460; doi:10.1371/journal.pone.0268792)
Supplement: S2 Table — (DOCX) [file pone.0268792.s002.docx]

**S2 Table. The prevalence of helminth eggs under the finger nails of 600 school children, Jimma Town (Ethiopia).**

|  | |  | **N** | | **Number of cases (%)** | | | | | | | | | | | | | |  | |  |
| --- | --- | --- | --- | --- | --- | --- | --- | --- | --- | --- | --- | --- | --- | --- | --- | --- | --- | --- | --- | --- | --- |
|  |  |  |  |  | Any STH | | *Ascaris* | | *Trichuris* | | Hookworm | | *Schistosoma* | | *Hymenolepis* | | *Taenia* | | *Enterobius* | |  |
| School | | | | | | | | | | | | | | | | | | |  | |  |
|  | 1 | | | 60 | | 5 (8.3) | | 5 (8.3) | | 1 (1.7) | | 0 | | 0 | | 1 (1.7) | | 1 (1.7) | | 0 | |
|  | 2 | | | 60 | | 0 | | 0 | | 0 | | 0 | | 0 | | 0 | | 0 | | 0 | |
|  | 3 | | | 60 | | 1 (1.7) | | 1 (1.7) | | 0 | | 0 | | 0 | | 0 | | 1 (1.7) | | 0 | |
|  | 4 | | | 60 | | 2 (3.3) | | 1 (1.7) | | 1 (1.7) | | 0 | | 0 | | 0 | | 1 (1.7) | | 0 | |
|  | 5 | | | 60 | | 2 (3.3) | | 1 (1.7) | | 1 (1.7) | | 0 | | 0 | | 0 | | 0 | | 0 | |
|  | 6 | | | 60 | | 0 | | 0 | | 0 | | 0 | | 0 | | 0 | | 1 (1.7) | | 3 (5.0) | |
|  | 7 | | | 60 | | 0 | | 0 | | 0 | | 0 | | 0 | | 0 | | 0 | | 0 | |
|  | 8 | | | 60 | | 2 (3.3) | | 2 (3.3) | | 0 | | 0 | | 0 | | 0 | | 1 (1.7) | | 0 | |
|  | 9 | | | 60 | | 0 | | 0 | | 0 | | 0 | | 0 | | 0 | | 0 | | 0 | |
|  | 10 | | | 60 | | 0 | | 0 | | 0 | | 0 | | 0 | | 0 | | 1 (1.7) | | 0 | |
| Sex | | | | | | | | | | | | | | | | | | |  | |  |
|  | Female | | | 313 | | 8 (2.6) | | 6 (1.9) | | 3 (1.0) | | 0 | | 0 | | 1 (0.3) | | 4 (1.3) | | 2 (0.6) | |
|  | Male | | | 287 | | 4 (1.4) | | 4 (1.4) | | 0 | | 0 | | 0 | | 0 | | 2 (0.7) | | 1 (0.3) | |
| Age group (years) | | | | | | | | | | | | | | | | | | |  | |  |
|  | 5 – 9 | | | 300 | | 9 (3.0) | | 9 (3.0) | | 1 (0.3) | | 0 | | 0 | | 1 (0.3) | | 3 (1.0) | | 3 (1.0) | |
|  | 14 – 18 | | | 300 | | 3 (1.0) | | 1 (0.3) | | 2 (0.7) | | 0 | | 0 | | 0 | | 3 (1.0) | | 0 | |
| **Total** | | | **600** | | **12 (2.0)** | | **10 (1.7)** | | **3 (0.5)** | | **0** | | **0** | | **1 (1.7)** | | **6 (1.0)** | | **3 (0.5)** | |  |
