# Supplementary material for: Assessment of the nail contamination with soil-transmitted helminths in schoolchildren in Jimma Town, Ethiopia
Source: PLoS One. 2022 Jun 29;17(6):e0268792. doi: 10.1371/journal.pone.0268792 (PMC9242460; doi:10.1371/journal.pone.0268792)
Supplement: S3 Table — (DOCX) [file pone.0268792.s003.docx]

**S3 Table.** **The personal hygiene practice among 600 school children from 10 governmental schools in Jimma Town (Ethiopia).**

| **Question** | | **Number of students (%)** |
| --- | --- | --- |
| ***Finger sucking habit*** | |  |
|  | Always | 6 (1.0) |
|  | Sometimes | 36 (6.0) |
|  | Never | 558 (93.0) |
| ***Trimming of nails*** | | |
|  | Yes | 580 (96.7) |
|  | No | 20 (3.3) |
| ***Frequency of trimming*** | | |
|  | 1x/week | 22 (3.7) |
|  | 1x/2weeks weeks | 237 (39.5) |
|  | 1x/3weeks | 7 (1.2) |
|  | When it is long enough | 315 (52.5) |
|  | No response | 19 (3.2) |
| ***Trimming material*** | | |
|  | Teeth | 17 (2.8) |
|  | Razor | 179 (29.8) |
|  | Nail clipper | 385 (64.2) |
|  | No response | 19 (3.2) |
| ***Washing hand before eating*** | | |
|  | Always | 557 (92.8) |
|  | Sometimes | 43 (7.2) |
|  | Never | 0 (0.0) |
| ***Hand washing frequency with soap before eating*** | | |
|  | Always | 273 (45.5) |
|  | Sometimes | 311 (51.8) |
|  | Never | 16 (2.7) |
| ***Hand washing after toilet*** | | |
|  | Always | 379 (63.2) |
|  | Sometimes | 221 (36.8) |
|  | Never | 0 (0.0) |
| ***Frequency of hand washing after toilet with soap*** | | |
|  | Always | 197 (32.8) |
|  | Sometimes | 364 (60.7) |
|  | Never | 39 (6.5) |
| ***Type of playing game on ground*** | | |
|  | Teter | 202 (33.7) |
|  | Kelbosh | 15 (2.5) |
|  | Segno maksegno | 36 (6.0) |
|  | Never played | 347 (57.8) |
| ***Untrimmed finger may be source of STH infection*** | | |
|  | Yes | 467 (77.8) |
|  | No | 52 (8.7) |
|  | I do not know | 81 (13.5) |
