# Supplementary material for: Assessment of the nail contamination with soil-transmitted helminths in schoolchildren in Jimma Town, Ethiopia
Source: PLoS One. 2022 Jun 29;17(6):e0268792. doi: 10.1371/journal.pone.0268792 (PMC9242460; doi:10.1371/journal.pone.0268792)
Supplement: S4 Table — This table summarizes the prevalence of soil-transmitted helminth infections (STH; Ascaris lumbricoides, Trichuris trichiura and hookworm) of any intensity and moderate-to-heavy intensity (MHI) across 3 cross-sectional surveys (2015, 2018 and 2020). In each survey, 600 different subjects from ten governmental schools were included. (DOCX) [file pone.0268792.s004.docx]

**S4 Table. The long-term impact of the national deworming program on STH infections in Jimma Town (Ethiopia).** This table summarizes the prevalence of soil-transmitted helminth infections (STH; *Ascaris lumbricoides*, *Trichuris* *trichiura* and hookworm) of any intensity and moderate-to-heavy intensity (MHI) across 3 cross-sectional surveys (2015, 2018 and 2020). In each survey, 600 different subjects from ten governmental schools were included.

|  | **Helminth** | **Infection intensity** | **School ID** | | | | | | | | | | **Total** |
| --- | --- | --- | --- | --- | --- | --- | --- | --- | --- | --- | --- | --- | --- |
|  |  |  | 1 | 2 | 3 | 4 | 5 | 6 | 7 | 8 | 9 | 10 |  |
| ***2015*** | | | | | | | | | | | | | |
|  | Any STH | Any | 60.0 | 58.3 | 60.0 | 55.0 | 61.7 | 53.3 | 45.0 | 58.3 | 58.3 | 66.7 | 57.7 |
|  |  | MHI | 10.0 | 15.0 | 20.0 | 13.3 | 16.7 | 10.0 | 5.0 | 8.3 | 15.0 | 10.0 | 12.3 |
|  | *Ascaris* | Any | 28.3 | 33.3 | 38.3 | 26.0 | 35.0 | 31.7 | 18.3 | 25.0 | 36.7 | 40.0 | 31.3 |
|  |  | MHI | 10.0 | 11.7 | 15.0 | 11.7 | 16.7 | 8.3 | 5.0 | 8.3 | 15.0 | 10.0 | 11.2 |
|  | *Trichuris* | Any | 40.0 | 35.0 | 55.0 | 40.0 | 45.0 | 31.3 | 28.3 | 36.7 | 41.7 | 41.7 | 39.5 |
|  |  | MHI | 0.0 | 3.3 | 6.7 | 5.0 | 1.7 | 1.7 | 0.0 | 1.7 | 5.0 | 0.0 | 2.5 |
|  | Hookworm | Any | 20.0 | 5.0 | 1.7 | 10.0 | 10.0 | 8.3 | 11.7 | 18.3 | 8.3 | 10.0 | 10.3 |
|  |  | MHI | 0.0 | 0.0 | 0.0 | 9.0 | 0.0 | 0.0 | 0.0 | 0.0 | 0.0 | 0.0 | 0.0 |
| ***2018*** | | | | | | | | | | | | | |
|  | Any STH | Any | 31.7 | 26.7 | 26.7 | 31.7 | 55.0 | 8.3 | 23.3 | 26.7 | 26.7 | 23.3 | 28.0 |
|  |  | MHI | 3.3 | 8.3 | 3.3 | 3.3 | 15.0 | 0.0 | 6.7 | 6.7 | 5.0 | 6.7 | 5.8 |
|  | *Ascaris* | Any | 11.7 | 21.7 | 6.7 | 10.0 | 28.3 | 3.3 | 15.0 | 18.3 | 6.7 | 11.7 | 13.3 |
|  |  | MHI | 3.3 | 8.3 | 3.3 | 3.3 | 13.3 | 0.0 | 6.7 | 6.7 | 5.0 | 3.3 | 5.3 |
|  | *Trichuris* | Any | 20.0 | 20.0 | 25.0 | 23.3 | 38.3 | 3.3 | 8.3 | 16.7 | 11.7 | 15.0 | 18.2 |
|  |  | MHI | 0.0 | 0.0 | 1.7 | 0.0 | 3.3 | 0.0 | 0.0 | 3.3 | 1.7 | 3.3 | 1.3 |
|  | Hookworm | Any | 11.7 | 0.0 | 0.0 | 3.3 | 5.0 | 3.3 | 5.0 | 0.0 | 16.7 | 5.0 | 5.0 |
|  |  | MHI | 0.0 | 0.0 | 0.0 | 0.0 | 0.0 | 0.0 | 0.0 | 0.0 | 0.0 | 0.0 | 0.0 |
| ***2020*** | | | | | | | | | | | | | |
|  | Any STH | Any | 25.0 | 25.0 | 30.0 | 18.3 | 26.7 | 35.0 | 18.3 | 25.0 | 20.0 | 20.0 | 24.3 |
|  |  | MHI | 6.7 | 8.3 | 0.0 | 1.7 | 1.7 | 10.0 | 3.3 | 8.3 | 1.7 | 3.3 | 4.5 |
|  | *Ascaris* | Any | 23.3 | 23.3 | 20.0 | 13.3 | 21.7 | 26.7 | 10.0 | 20.0 | 15.0 | 11.7 | 18.5 |
|  |  | MHI | 6.7 | 8.3 | 0.0 | 0.0 | 1.7 | 10.0 | 3.3 | 8.3 | 1.7 | 3.3 | 4.3 |
|  | *Trichuris* | Any | 6.7 | 8.3 | 15.0 | 6.7 | 15.0 | 8.3 | 8.3 | 10.0 | 11.7 | 8.3 | 9.8 |
|  |  | MHI | 0.0 | 0.0 | 0.0 | 1.7 | 0.0 | 0.0 | 0.0 | 0.0 | 0.0 | 0.0 | 0.2 |
|  | Hookworm | Any | 0.0 | 0.0 | 0.0 | 0.0 | 0.0 | 1.7 | 1.7 | 0.0 | 1.7 | 0.0 | 0.5 |
|  |  | MHI | 0.0 | 0.0 | 0.0 | 0.0 | 0.0 | 0.0 | 0.0 | 0.0 | 0.0 | 0.0 | 0.0 |
