# Supplementary material for: Assessment of the nail contamination with soil-transmitted helminths in schoolchildren in Jimma Town, Ethiopia
Source: PLoS One. 2022 Jun 29;17(6):e0268792. doi: 10.1371/journal.pone.0268792 (PMC9242460; doi:10.1371/journal.pone.0268792)
Supplement: S1 File — (DOCX) [file pone.0268792.s006.docx]

**Assessment of the nail contamination with soil-transmitted helminths and the impact of the 5 rounds of biannual deworming on infections in school children in Jimma Town, Ethiopia**

Bamlaku Tadege^1,2,3*^, Zeleke Mekonnen^2^, Daniel Dana^2,3^, Abebaw Tiruneh^2^, Bizuwarek^2^ Sharew^2^, Eden Dereje^4^, Eskindir Loha^5,6^, Mio Ayana^2,3^ and Bruno Levecke^3*^

^1^School of Medical Laboratory Sciences, Hawassa University, Hawassa, Ethiopia; ^2^School of Medical Laboratory Sciences, Jimma University, Jimma, Ethiopia; ^3^Department of Virology, Parasitology and Immunology, Ghent University, Merelbeke, Belgium; ^4^Molecular Biology and NTDs Research Center, Jimma University, Jimma, ^5^Chr. Michelsen Institute, Bergen, Norway; ^6^Centre for International Health, University of Bergen, Bergen, Norway

*e-mail corresponding authors: [abelbamlaku@yahoo.com](mailto:abelbamlaku@yahoo.com) and [bruno.levecke@ugent.be](mailto:bruno.levecke@ugent.be)

**S1 Info. The English version of the questionnaire on personal hygiene practices**

**School name ----------------------- ID ------------------ grade --------- mobile -----------------**

**Maqaa mana barumsaa_________________lakk.___________kutaa_______lakkofsa bilbilaa________**

**Demographic and personal hygiene characteristics of school children in Jimma Town elementary schools**

| **S.NO** | **Characteristics= amala** | **Category= garee** |
| --- | --- | --- |
| 1 | Age = umurii |  |
| 2 | Sex= saala | 01 = Male= dhiira  02 = Female= dubara |
| 3 | Grade = kutaa |  |
| 4 | Do you have nail suck habit?  Amala quba kee afaan keessa kaahuu qabdaa? | 01 = Always= yeroo hunda  02 = Sometimes= takka takka  03= Never = tasuma ( lakki) |
| 5 | Do you trim your nails periodically?  Haala itti fufinsa qabuun qeense kee ni qorattaa? | 01= yes= eeyyee  02= Sometimes= takka takka  03= Never= lakki |
| 5a | If you trim your nails periodically for Q5 how often do you trim?  Yoo deebiin kee gaaffii lakkoofsa 5ffaaf eeyyee ta’e, garaagarummaa yeroo hangamiin qorattaa? | 01= One time per week = torbanitti takkaa  02 = One time per two weeks = torban lamatti takkaa  03 = One time per three weeks and above= torbaan sadiitti ykn isaa olitti takkaa  04 = when it is big enough= yeroo inni sirritti guddatu |
| 5b | If you trim your nails, for Q5 what do you use to trim you nails?  Qeensa kee ni qoratta yoo ta’e 5(a)ffaa, maaliin qorattaa? | 01= using my own teeth = ilkaaniin  02 = razor= millaaccii  03 = nail clipper = kuttuu qeensaa |
| 6 | Do you wash your hands before eating?  Nyaata dura harka kee ni dhiqattaa? | 01= Always= yeroo hunda  02= Sometimes= takka takka  03= Never= hin dhiqadhu |
| 6a | If you wash your hand for Q6 do you use soap?  Yoo deebiin gaaffii 6ffaa na dhiqadha ta’e, samunaa ni fayyadamtaa? | 01= Always= yeroo hunda  02= Sometimes= takka takka  03= Never= hin fayyadamu |
| 7 | Do you wash your hands after going to the toilet?  Mana fincaanii fayadamtee yeroo deebitu harka kee ni dhiqataa? | 01= Always= yeroo hundaa  02= Sometimes= takka takka  03= Never= hin dhiqadhu |
| 7a | If you wash your hand for Q7 do you use soap?  Yoo deebiin kee lakkofsa 7ffaaf eeyyee harka koo nan dhiqadhadha ta’e, saamunaa ni fayadamtaa? | 01= Always= yeroo hundaa  02= Sometimes= takka takka  03= Never= hin fayyadamu |
| 8 | Which game do you play on the ground(soil)? More than one answer possible  Tapha lafarraa keessaa isa kam taphatta? Deebii tokkoo ol kennuun ni danda’ama | 01= marble(biy)= biyyii  02= kelebosh=  03= Teter= xaxarii  04= Gebetta= saddeeqa  05= Segno maksegno= wixataa fi kibxata  06= Never play= tasa hin taphadhu |
| 9 | Untrimmed nail can be a source of intestinal parasite infection  Qeensi hin qoramne madda raammoo maxxantuu garaaf nama saaxila | 01= yes= eeyyee  02= no= lakki  03= I do not know= Ani hin beeku |

Data collector Name -------------------------- signature ----------------- date-------------

Maqaa nama ragaa funaanee_________ mallattoo_____________guyyaa_____
